# Knowledge, attitude, and perception on COVID-19 vaccine acceptance: cross-sectional study among Eritrean refugees in Kampala

**DOI:** 10.11604/pamj.2024.49.103.44553

**Published:** 2024-12-02

**Authors:** Robel Tesfahiwet Enghida, Tesfaldet Habtemariam Hidru

**Affiliations:** 1 Cavendish University Uganda, Kampala, Uganda; 2 Dalian Medical University, Dalian, China

**Keywords:** COVID-19, vaccine acceptance, refugees, knowledge

## Abstract

**Introduction:**

the impact of Coronavirus disease (COVID-19) on public health has been significant and wide-ranging. It has led to widespread illness, strain on healthcare systems, and various public health measures to control its spread. Several pharmaceutical companies, researchers, and scientists have been racing against the clock to develop a safe and effective vaccine against COVID-19 in a short period. Despite the efforts by governments, organizations, and public health experts to reach the target population, vaccine hesitancy among the communities remains a major impediment to mitigating the COVID-19 pandemic. A strategy to address the factors that contribute to low vaccine utilization must be in place for a vaccination program to achieve herd immunity.

**Methods:**

a cross-sectional descriptive survey of 383 study participants was conducted between February 26^th^ and March 24^th^, 2022. A multistage sampling technique was used to enroll the study. For subjects, data was collected by trained research assistants who speak Eritrea's native language (Tigrigna) using a pretested structured questionnaire. The factors associated with the acceptance of the COVID-19 vaccine were identified using a binary logistic regression model at P-values less than 0.05.

**Results:**

according to the findings of this study, 65.8% of Eritrean refugees in Kampala were willing to accept the COVID-19 vaccine. The study discovered that marital status, knowledge of COVID-19, positive attitude toward the COVID-19 vaccine, and belief in conspiracy theories are the independent factors that are associated with vaccine acceptance behavior among refugees.

**Conclusion:**

there was a low likelihood of accepting the COVID-19 vaccine in this study. Thus, stakeholders must work together to educate the public about the COVID-19 vaccine. The health authority must inform the public about the vaccine's benefits while also addressing the vaccine's side effects and safety concerns.

## Introduction

The impact of Coronavirus disease (COVID-19) on public health has been significant and wide-ranging. It has led to widespread illness, strain on healthcare systems, and various public health measures to control its spread [1]. These include lockdowns, social distancing, mask mandates, and vaccination campaigns [1,2]. The pandemic has also highlighted the importance of preparedness and international cooperation in managing global health crises. Following the outbreak, researchers have been racing against the clock to develop a safe and effective vaccine against COVID-19 in a short period. As a result, the World Health Organization (WHO) approved several vaccines against COVID-19 for emergency use to decrease the morbidity and mortality caused by the disease [3]. However, the success rate of vaccine coverage has varied across countries.

An estimated 11.6 billion COVID-19 vaccine doses have been delivered worldwide (as of March 2^nd^, 2022), with approximately 58% of the population completely immunized [4]. This indicates that the acceptance rate of the COVID-19 vaccine seems concerning though the vaccine coverage may vary across nations. For instance, a systematic analysis of 60 studies from 33 countries found that the COVID-19 acceptance rates were below 60%. Notably, the Middle East, Russia, Africa, and several European nations have all reported poor rates of COVID-19 vaccine adoption [5]. These shreds of evidence revealed a need for a thorough assessment of factors associated with a low rate of COVID-19 vaccination acceptance.

Vaccination programs require a proper strategy of vaccine accessibility to the entire community. Despite the efforts by governments, organizations, and public health experts to reach the target population, vaccine hesitancy among communities remains a major obstacle to containing the COVID-19 pandemic [6]. In Uganda, 14.5 million doses of COVID-19 vaccine were given out, with around 8 million persons receiving complete immunization [7-9]. According to research conducted in western Uganda in 2020, before the rollout of the vaccination, 53.6% of people were eager to embrace the COVID-19 vaccine [10]. Indeed, this low acceptance rate could negatively influence the attempt to reach the target population and contain the pandemic. Interestingly, Uganda is a home of many immigrants [11], therefore, it is imperative to develop or align national COVID-19 vaccination programs with humanitarian agencies to increase the uptake of COVID-19 vaccine [12].

The COVID-19 vaccine utilization is expected to reduce COVID-19 severity. To enhance vaccination coverage, it is necessary to understand the obstacles that lead to vaccine reluctance. Vaccine acceptability may be associated with one's cultural background, beliefs, knowledge, attitudes, perception, personal, political, and environmental factors [13,14]. Currently, there is a large body of evidence on COVID-19 vaccine availability and distribution. However, vaccine acceptability, an important domain that determines vaccine uptake and control of infection, is gaining momentum. So far, there is limited evidence of vaccine acceptability among humanitarian populations in low-income countries. And, to the extent of our knowledge, there is no study on COVID-19 vaccine acceptability among Eritrean refugees in Uganda. Therefore, this study aimed to identify factors that are associated with COVID-19 vaccine acceptance among Eritrean refugees in Uganda.

## Methods

**Study design:** a cross-sectional study was conducted to identify factors associated with COVID-19 vaccine acceptability among Eritrean refugees in Kampala, Uganda.

**Study setting:** the study was conducted in Kampala, the capital city of Uganda, an East African country recognized as Africa's largest refugee-hosting nation [11]. Kampala is a bustling urban area divided into five administrative divisions: Central, Kawempe, Makindye, Nakawa, and Rubaga. The city is a major hub for refugees, including those from Eritrea. The environment of Kampala, with its unique socio-economic conditions, infrastructure, and resources, provided the backdrop for this study. Data was collected within this urban setting between 26^th^ February and 24^th^ March 2022.

**Study population:** the study focused on adult Eritrean refugees residing in Kampala. According to the United Nations High Commissioner for Refugees (UNHCR) and the Office of the Prime Minister of Uganda (OPM), as of 30th June 2021, there were approximately 1.5 million refugees in Uganda, with more than 33,000 Eritreans living in Kampala [11]. The study targeted Eritrean refugees of 18 years and above who had registered with the authorities between January 2016 and December 2021. Only those with valid refugee identification cards were eligible. The study excluded individuals who were mentally ill, seriously ill, or residing in other parts of Uganda.

**Sample size:** a single proportion formula was used to determine the sample size.


$$\text{n = }\frac{{\text{Z}}^{\text{2}}\text{×P×(1-P)}}{{\text{d}}^{\text{2}}}$$

Where, Z^2^ = value at 95% confidence interval is always 1.96, P=prevalence of 50% for the proportion of population accepting the COVID-19 vaccine was taken since there is no similar study in the study area. d^2^ is the margin of error (5%).

Therefore, 383 respondents were selected using a multistage cluster sampling method based on the population's geographical location. A simple random sampling technique was used to choose parishes from each sub-county in the Kampala district. The eligible individuals from the designated parishes were recruited using a systematic random sampling technique. The sampling frame was based on the Uganda Bureau of Statistics and the UNHCR (OPM) ProGres database of all registered Eritrean refugees and asylum seekers in Kampala, which comprises eligible people listed by name, age, address, and contact information. The involvement of community leaders and local councils was sought to facilitate the process.

**Variables:** this research was guided by the following variables. The COVID-19 vaccine acceptance, defined as a decision to accept (including the vaccinated) or refuse, when presented with an opportunity, is the dependent variable of the study. The primary outcome will be acceptance or refusal of COVID-19 vaccination among the refugees. The factors associated with acceptance of the COVID-19 vaccine considered as independent variables include socio-demographic factors, knowledge, attitude towards the vaccine, conspiracy theory, health education and previous vaccine experience. These variables are mostly categorical.

### Data resource and measurement

***Data collection tool:*** in this study, we designed a new instrument [attitude and conspiracy on COVID-19 Vaccine Acceptance (KACC-19VA score)] based on the frequently included questions in other studies that deal with vaccine acceptance [15,16]. It was pre-tested to verify whether it could accurately assess the acceptance of the COVID-19 jab within the Eritrean community. The KACC-19VA was used as part of a composite questionnaire to assess the knowledge and awareness, vaccine attitudes, and conspiracy among study participants. The tool was translated into Tigrigna and was scored using a four-point scale (1 to 4). Higher scores indicate a higher tendency of COVID-19 acceptance. The internal consistency of the questionnaire was evaluated using Cronbach's alpha coefficient, which was 0.71, 0.74, and 0.72 for knowledge, attitude toward vaccines, and hesitancy/conspiracy, respectively. Cronbach's alpha greater than 0.6 is regarded as an adequate and accurate measurement [17].

***Data collection:*** participants were interviewed using a structured questionnaire to obtain demographic data, personal attributes (factors that influence COVID-19 vaccination intention), and healthcare-related factors that are associated with the acceptance of vaccines. Public health professionals, trained specifically to collect the data, were involved in administering the questionnaires. Vaccine acceptance behavior was defined as a person's willingness to accept or refuse the COVID-19 vaccination, and it was assessed subjectively by asking the participants about their agreement and disagreement (1 = yes, 0 = no) with a question that assesses if they intend to take COVID-19 vaccine.

***Data analysis:*** statistical analysis IBM-SPSS software, V.26, was used for the statistical analysis. Normally distributed continuous variables were presented as the mean ± standard deviation (SD) whereas the non-normally distributed data were expressed as median (interquartile range). The chi-square test was applied to compare different groups of categorical variables and output was summarized using counts and percentiles. The difference among the continuous variables between acceptance and non-acceptance groups was tested using independent t-tests. Spearman rank order correlation was used to explore correlates of vaccine acceptance. We run binary logistic regression analysis to assess the predictors of vaccine acceptance. Preliminary analyses were conducted to ensure no violation of the assumptions of normality, linearity, multicollinearity, and homoscedasticity. We executed receiver operating characteristics (ROC) to evaluate the predictive performance of the KACC-19VA Score. A two-sided P-value of 0.05 was considered statistically significant.

**Ethical consideration:** the researcher had obtained permission to conduct the study from Cavendish University Uganda, Faculty of Health Sciences Research and Ethics Committee (CUU-REC). Informed consent was obtained from the respondents by writing a statement at the top of the questionnaire and only those that agreed to consent participated in the study. The consent form described the purpose of the study, the procedures to be followed, the risks and benefits of participation as well as the rights of the participants. Participants were informed that their participation was purely voluntary and that they could withdraw from the study at any time. They were informed that choosing not to participate in the study would not impact their studies. Data collected from the participants were handled by the researchers and kept in securely locked cabinets and on a password-protected computer. The names of the study participants never appeared on the study form, instead an identification number was used.

## Results

**Socio-demographic and clinical characteristics:** the baseline characteristics of the participants are summarized in Table 1. In the total 383 participants, 173 (45.17%) were males and 210 (54.83%) were females. Of these, 252 (65.8%) participants were willing to accept the COVID-19 vaccine whereas 131 (34.2%) were not. The median (25-75%) for KACC-19VA score in acceptance and non-acceptance groups were 25 (23-27) and 20 (17-22), respectively. The participants in the vaccine acceptance group were more likely to be educated, with a significantly higher knowledge score (P<0.001), attitude towards vaccine score (P < 0.001), and KACC-19VA score (P<0.001). Moreover, married participants (X^2^=227.128, P<0.001) and those who were exposed to health education (X^2^=13.376, P<0.001) were more willing to accept the COVID-19 vaccine than those who did not accept COVID-19 vaccination. However, the conspiracy score was higher in the non-acceptance group (P<0.001).

**Table 1:** demographic characteristics of the Eritrean refugee participants (n=383) in Kampala, Uganda, collected in March 2022

Variable	Not Accepted (n=131)	Accepted (n=252)	x^2^/t	P-Value
**Age**				
18-27	61 (46.6%)	90 (35.7%)	7.977	0.092
28-37	50(38.2%)	99 (39.3%)		
38-47	16 (12.2%)	43 (17.1%)		
>49	4 (3.1%)	20(8.0%)		
**Sex**				
Female	65 (49.6%)	145 (57.5%)	2.184	0.139
**Marital status**				
Single	85 (64.9%)	93 (36.9%)	27.128	<0.001
**Educational level**				
Non-formal	25 (19.1%)	8 (3.2%)	45.309	<0.001
Primary	45.8%	88 (34.9%)		
Secondary	45 (34.4%)	128 (50.8%)		
Post-secondary	1 (0.8%)	28 (11.1%)		
**Income**				
Employment, yes	43 (32.8%)	75 (29.8%)	.379	0.538
**Availability of nearby vaccination center, yes**	30 (22.9%)	82 (32.5%)	3.870	0.049
**Health education exposure**	37 (28.2%)	120 (47.6%)	13.376	<0.001
**Bad vaccine experience**	5 (3.8%)	10 (4.0%)	.005	0.942
**Attitude towards vaccine**	7 (6-9)	11 (9-12)	10.256	<0.001
**Conspiracy theory**	4.40±1.77	2.66±1.80	9.006	<0.001
**Knowledge**	7.00 (6.0-10.0)	12.00 (10.0-12.0)	11.769	<0.001
**KACC-19VA score**	20 (17-22)	25 (23-27)	10.596	<0.001

**Note:** KACC-19VA, Score for knowledge, attitude and conspiracy on COVID-19 Vaccine Acceptance

**Correlates of COVID-19 vaccine acceptance:** Table 2 illustrates the correlation between socio-demographic characteristics and COVID-19 vaccine acceptance. Vaccine acceptance was significantly correlated with age (P=0.010), marital status (P<.001), educational level (P<.001), nearby vaccination service availability (P=0.049), health education exposure (P<.001), knowledge score (P<.001), attitude towards vaccine score (P<.001), and KACC-19VA Score (P<.001). In addition, vaccine acceptance was negatively correlated with conspiracy score (P<.001).

**Table 2:** correlates of COVID-19 vaccine acceptance among Eritrean refugee participants (n=383) in Kampala, Uganda

Variable	Spearman Correlation (r)	P-Value
Age	0.131*	0.010
gender	-0.076	0.140
Marital status	0.266**	<.001
Educational status	0.316**	<.001
Income		
Employment	-0.031	0.539
Nearby vaccination service availability	0.101*	0.049
Health education exposure	.187**	<.001
Bad vaccine experience	0.004	0.931
Knowledge	0.622**	<.001
Attitude towards vaccine	0.512**	<.001
Conspiracy theory	-0.419**	<.001
KACC-19VA score	0.564**	<.001

Note: KACC-19VA, Score for knowledge, attitude and conspiracy on COVID-19 vaccine acceptance

**Predictors of COVID-19 acceptance:** Table 3 presents the binary logistic regression analysis for influential factors associated with COVID-19 vaccine acceptance. We observed a positive relationship between marital status and vaccine acceptance. Married participants had a higher likelihood of vaccine acceptance compared with single ones. The OR and 95% CI for married participants were 3.619 (95% CI: 1.56-8.398, P=0.003). Likewise, compared to informal education, the primary (OR=9.718; 95% CI: 2.089-45.22, P=0.004)], secondary (OR=12.848; 95% CI: 2.804-58.859, P<.001), and post-secondary (OR=16.201; 95% CI: 5.78-20.194, P<.001) educational levels were associated with higher acceptability of COVID-19 vaccination. Also, the regression model showed that knowledge (OR: 2.015; 95% CI: 1.686-2.407, P<.001) and attitude towards vaccine (OR: 1.574; 95% CI: 1.337-1.853, P<.001) scores were independently associated with vaccine acceptance. We observed that participants with high scores of conspiracies (OR: 0.753; 95% CI: 0.601-0.943, P=0.014) had a lower likelihood of vaccine acceptance.

**Table 3:** predictors of COVID-19 acceptance among Eritrean refugee participants (n=383) in Kampala, Uganda

Variables	B	S.E.	Wald	OR (95% C.I)	P-Value
**Age (18-27)**			5.725		0.221
28-37	0.125	0.451	0.076	1.133 (0.468-2.743	0.783
38-49	-1.163	0.602	3.732	0.313 (0.096-1.017	0.053
>49	-0.887	0.943	0.884	0.412 (0.065-2.617)	0.347
**Sex**					
**Male**	-0.268	0.425	0.400	0.765 (0.333-1.757)	0.527
**Marital status**					
**Married**	1.286	0.429	8.971	3.619 (1.56-8.398)	0.003
**Education, (informal)**			14.243		0.003
Primary	2.274	0.784	8.403	9.718 (2.089-45.22)	0.004
Secondary	2.553	0.777	10.810	12.848 (2.804-58.859)	0.001
Post-secondary	4.333	1.316	10.841	16.201 (5.78-20.194)	0.001
**Income**					
Employment	-0.200	0.473	0.178	0.819 (0.324-2.069)	0.673
**Availability of nearby health service**	0.276	0.444	0.386	1.317 (0.552-3.144)	0.534
**Health Education exposure**	-0.136	0.413	0.108	0.873 (0.389-1.960)	0.742
**Bad vaccine experience**	-0.455	0.882	0.266	0.634 (0.113-3.574)	0.606
**Attitude towards vaccine**	0.454	0.083	29.686	1.574 (1.337-1.853)	0.001
**Conspiracy theory**	-0.284	0.115	6.079	0.753 (0.601-0.943)	0.014
**Knowledge**	0.701	0.091	59.501	2.015 (1.686-2.407)	<.001
**Constant**	-12.047	1.760	46.850	0.000	<.001

The model was adjusted for age, sex, marital status, educational level, employment status, availability of nearby health service, health Education exposure, bad vaccine experience, attitude towards vaccine, conspiracy theory, knowledge. B: Estimated coefficient, SE: Standard error, Wald: ratio of B to SE, OR: odds ratio

**The predictive performance of KACC-19VA:** further, we performed receiver-operating characteristic (ROC) analysis to determine the predictive capacity of KACC-19VA to identify participants' willingness to accept COVID-19 vaccination. The AUC for the KACC-19VA model was observed to be 0.829 (95% CI: 0.782- 0.875), suggesting a positive predictive capacity. The results for the area under the ROC Curve are provided in Table 4.

**Table 4:** area under the ROC curve for predictors of COVID-19 vaccine acceptance among Eritrean refugee participants (n=383) in Kampala, Uganda

				Asymptotic 95% confidence Interval	
Test result variable (s)	Area	Std. Errora	Asymptotic Sig.b	Lower bound	Upper bound
KACC-19VA score	.829	.024	.000	.782	.875

Note: KACC-19VA Score; knowledge, attitude towards vaccine, conspiracy on COVID-19 Vaccine acceptance. ROC: Receiver Operating Characteristic

## Discussion

In this study, a significant proportion (34.2%) of Eritrean refugees in Kampala were not willing to accept COVID-19 vaccines. Vaccine acceptance was significantly associated with marital status, educational level, knowledge score, attitude towards vaccine score, and KACC-19VA score. In addition, vaccine acceptance was negatively correlated with conspiracy scores. According to the findings of our study, 65.8 percent of Eritrean refugees in Kampala responded that they were willing to accept COVID-19 vaccination. Akin to the previous studies that were conducted among adults in Ethiopia (62.6%) and Ireland (65%) [18,19], the intention to receive the COVID-19 vaccination in this study was modest. Besides, this study showed a higher level of acceptance of the vaccine compared to the studies conducted in South Africa (48%), Zimbabwe (50%) [20], and Pakistan (47%) [21].

A recent study among Ugandan medical students reported that the vaccine acceptance rate was only 37.3%, almost lower by 50% compared to our findings. However, studies among the high-risk group in Uganda (70.1%), Cameroon (84.6%), Australia (89.88%), the United Kingdom (71%), and the United States (69%) found a higher rate compared to our study [22,23]. This difference can be explained by disparities in the respondents' socio-demographic characteristics, the availability and accessibility of infrastructures, and understanding of the host country's health system. Participants with higher levels of education had shown increased knowledge of the COVID-19 disease which was positively correlated with the acceptance of the COVID-19 vaccine. Similar findings were found in the UK, Australia, and some Middle Eastern populations. These studies found a positive correlation between education level and readiness to accept the COVID-19 vaccine, whereas a study from Greece reported a negative correlation [24-26]. However, cross-sectional studies from Kenya and Uganda have shown no significant relationship between educational levels and COVID-19 vaccine acceptance [27,28].

This study illustrates that marital status was one of the independent predictors of COVID-19 vaccine acceptability. Married participants were twice as likely as their single counterparts to accept the COVID-19 vaccination. The study showed similar results to studies done in Saudi Arabia among the general population [29]. Similarly, other studies conducted in Japan and Kuwait found that married people were more likely to accept the vaccine than unmarried people [30,31].

According to the findings of this study, believing in these conspiracy theories is one of the independent factors that hurt vaccination acceptance. In the past, many participants in a survey of healthcare workers in the Democratic Republic of the Congo believed the virus was created to diminish the world's population [31]. Besides, the anti-vaccination movement has grown due to various conspiratorial theories spread via social media platforms. These messages include the vaccine's association with mercury content, concerns regarding adverse effects of vaccine side effects and related safety issues, a lack of trust in the process, and the link between autism and infertility [28].

**Limitations:** the study populations are derived from refugees of a single nationality. Therefore, the finding may not be generalizable to all refugees in Kampala, but it offers important information on future vaccine uptake approaches among refugees. Secondly, the study was based on self-reported data, which might be influenced by recall or social desirability bias.

## Conclusion

According to the findings of this study, 65.8% of Eritrean refugees in Kampala were going to accept the COVID-19 vaccine and 13.1% had been vaccinated so far. This shows the vaccination behavior of the refugees is low and needs to be addressed to reach the vaccination target for herd immunity. The study revealed that socio-demographic characteristics such as having children and marital status are connected to the intention to accept the COVID-19 vaccine. Marital status is an independent predictor of COVID-19 acceptance. The study also showed knowledge of COVID-19, positive attitude toward the COVID-19 vaccine, and belief in conspiracy theories to be independent predictors of vaccine acceptance behavior. Health-related factors like trust in the health system, negative information and advice from healthcare workers were also factors that were associated with COVID-19 vaccine acceptance among Eritrean refugees in Kampala.

### What is known about this topic

- Vaccines, which have contributed to eradicating certain diseases, are the most effective means of disease prevention;
- The major obstacles to successfully implementing COVID-19 vaccination programs to combat the unprecedented pandemic included vaccine mass manufacturing, equitable global distribution, uncertainty about long-term efficacy and vaccine refusal.

### What this study adds

- The study provides detailed data on the prevalence and predictors of COVID-19 vaccine acceptance specifically among Eritrean refugees in Kampala, Uganda;
- This study highlights significant predictors of vaccine acceptance and identifies key demographic factors that correlate with higher or lower acceptance rates within this refugee population; these insights are crucial for developing targeted public health interventions and strategies to increase vaccine uptake in similar refugee settings;
- Offers localized evidence on vaccine acceptance in a context with limited prior research, contributing to a better understanding of vaccine hesitancy and acceptance in refugee communities.
